# Spatial distribution and influencing factors of CDC health resources in China: a study based on panel data from 2016–2021

**DOI:** 10.3389/fpubh.2024.1331522

**Published:** 2024-05-01

**Authors:** Yingying Yu, Jiachen Lu, Xiaofeng Dou, Yaohui Yi, Ling Zhou

**Affiliations:** School of Public Health, Dalian Medical University, Dalian, China

**Keywords:** healthcare resources, spatial autocorrelation, spatial econometric model, influential factors, China

## Abstract

**Background:**

Measuring the development of Chinese centers for disease control and prevention only by analyzing human resources for health seems incomplete. Moreover, previous studies have focused more on the quantitative changes in healthcare resources and ignored its determinants. Therefore, this study aimed to analyze the allocation of healthcare resources in Chinese centers for disease control and prevention from the perspective of population and spatial distribution, and to further explore the characteristics and influencing factors of the spatial distribution of healthcare resources.

**Methods:**

Disease control personnel density, disease control and prevention centers density, and health expenditures density were used to represent human, physical, and financial resources for health, respectively. First, health resources were analyzed descriptively. Then, spatial autocorrelation was used to analyze the spatial distribution characteristics of healthcare resources. Finally, we used spatial econometric modeling to explore the influencing factors of healthcare resources.

**Results:**

The global Moran index for disease control and prevention centers density decreased from 1.3164 to 0.2662 (*p* < 0.01), while the global Moran index for disease control personnel density increased from 0.4782 to 0.5067 (*p* < 0.01), while the global Moran index for health expenditures density was statistically significant only in 2016 (*p* < 0.1). All three types of healthcare resources showed spatial aggregation. Population density and urbanization have a negative impact on the disease control and prevention centers density. There are direct and indirect effects of disease control personnel density and health expenditures density. Population density and urbanization had significant negative effects on local disease control personnel density. Urbanization has an indirect effect on health expenditures density.

**Conclusion:**

There were obvious differences in the spatial distribution of healthcare resources in Chinese centers for disease control and prevention. Social, economic and policy factors can affect healthcare resources. The government should consider the rational allocation of healthcare resources at the macro level.

## Introduction

Adequate healthcare resources are the cornerstone of a functioning health system and a prerequisite for ensuring that effective health services are available to different groups (1, 2). Healthcare resources allocation refers to the manner in which healthcare resources flow between health care institutions (sectors) or regions, and can reflect the level of health services (2). Healthcare resources affect the residents’ access to health care services, and studies show a positive correlation between healthcare resources and access to health care (3, 4). To optimize the allocation of healthcare resources in rural areas and contribute to more convenient access to health services for local residents (4). Rational healthcare resources allocation helps people achieve more desirable health outcomes (5, 6). For example, it has been found that adequate healthcare resources are conducive to reducing child COVID-19 mortality and morbidity (7). Health professionals are able to provide quality health services to tuberculosis patients, improving their quality of life and health outcomes (8).

The uneven distribution of healthcare resources is a long-standing challenge in China. It is also prevalent in other countries (regions), such as the United States (9), Portugal (10), Japan (11), Kenya (12), and Southeast Asia (13). As the largest developing country with a vast land area and a large population, China faces the challenge of maximizing the provision of comprehensive health care services given the constraints on available healthcare resources (14). A number of studies had evaluated healthcare resources allocation in China, but focused more on the entire health care system (15), Traditional Chinese Medicine (14), maternal and child health care (16), and primary health care system (17). Little attention is paid to the disease prevention and control system.

Chinese Centers for Disease Control and Prevention (CCDC) are an important part of China’s public health system. It has a mission to create a healthy and safe living environment, maintain social stability and promote people’s health by preventing and controlling disease, injury and disability (18). CCDC began in the 1940s and has been developing for more than 70 years. Nowadays, a unique four-tier disease prevention and control system has been formed, which contains national, provincial, municipal and county-level disease prevention and control centers (19). In 2016, the Healthy China 2030 plan emphasized adherence to prevention as the mainstay and prevention and control of major diseases, highlighting the importance of the CCDC in promoting the process of Healthy China (20). In 2019, the COVID-19 outbreak gave the public a more direct understanding of CCDC’s critical role in public health incident response. After the epidemic, the government of China accelerated the development and construction of the disease prevention and control system, with particularly focusing on optimizing the allocation of healthcare resources in regional CCDC (21). In 2023, the State Council of China issued the Guiding Opinions on Promoting the High-Quality Development of the Disease Prevention and Control Business, which particularly emphasized the necessity of promoting the reform of the CCDC system and building a strong public health system (22).

Healthcare resources affect the capacity and capability of CCDC to provide preventive health services (23). Health workforce is the most dynamic and critical element of the CCDC system, determining the level and quality of health services and influencing citizens’ opportunities to access preventive care (24). Health material resources are the essential condition for the provision of preventive health services to citizens and the basic indicator of the level of public health service. Health financial resources represent the impact of healthcare resources allocation on the health of the population (25). In summary, healthcare resources are crucial to the development of CCDC. Adequate healthcare resources will not only be conducive to promoting the high-quality development of CDC, but will also facilitate the achievement of the strategic goal of Healthy China. Therefore, it is necessary to conduct a detailed study of CCDC healthcare resources.

Currently, scholars mostly use equity analysis methods to analyze healthcare resources, such as the Gini coefficient, the Theil index and the Health Resource Density Index(HRDI), etc. (26). Ao et al. used the Gini coefficient, the Theil index and HRDI to evaluate the equity of rural healthcare resources in China (27). Jian sun used the concentration index to analyze the equity in the distribution of health materials and health human resources (28). Liu et al. used the Gini coefficient combined with spatial autocorrelation to analyze the inequality of healthcare resources in Traditional Chinese Medicine hospitals (29). These methods analyzed equity in the regional allocation of healthcare resources only in terms of population and geographic area. Although spatial autocorrelation analysis was applied, the distribution of healthcare resources was not spatially analyzed in depth. In addition, most of the studies on CCDC only used descriptive analysis methods such as rates and composition ratios to make a simple quantitative analysis of human resources for health (30–32). Fewer studies have addressed financial and material resources for health and have not been analyzed using more specialized statistical methods.

Based on panel data from 2016 to 2021, this study aims to use spatial autocorrelation analysis to explore the characteristics of the distribution of healthcare resources in CCDC and use spatial econometric models to assess the factors influencing health resources in CCDC from four major aspects: social, economic, health, and policy. To provide a theoretical basis for the health department to carry out the next health system reform and to gain a more comprehensive understanding of the factors affecting the development of the CDC from multiple perspectives.

## Materials and methods

### Data sources and variable selection

#### Data sources

This study used panel data for 31 provinces in China from 2016 to 2021. Hong Kong, Macau and Taiwan provinces are not included because of inconsistent statistical standards for the data. All data are from the China Health and Family Planning Statistical Yearbook, China Health Statistical Yearbook and China Statistical Yearbook.

#### Healthcare resources indicators

Based on the configuration of the population served and with reference to other studies, the number of disease control personnel per 10,000 population (disease control personnel density), the number of CCDC per 10,000 population (CCDC density), and *per capita* health expenditures (health expenditures density) were selected as indicators of healthcare resources in this study (33–36). The calculation method is as follows.


$$\mathrm{Disease}\;\mathrm{control}\;\mathrm{personnel}\;\mathrm{density}=\frac{\mathrm{Number}\;\mathrm{of}\;\mathrm{disease}\;\mathrm{control}\;per\mathrm{sonnel}}{\mathrm{Population}\;\mathrm{at}\;\mathrm{the}\;\mathrm{end}\;\mathrm{of}\;\mathrm{the}\;\mathrm{year}}$$

$$\mathrm{CCDC}\;\mathrm{density}=\frac{\mathrm{Number}\;\mathrm{of}\;\mathrm{CCDC}}{\mathrm{Population}\;\mathrm{at}\;\mathrm{the}\;\mathrm{end}\;\mathrm{of}\;\mathrm{the}\;\mathrm{year}}$$

$$\mathrm{Health}\;\mathrm{expenditures}\;\mathrm{density}=\frac{\mathrm{Health}\;\mathrm{expenditures}\;\mathrm{of}\;\mathrm{CCDC}}{\mathrm{Population}\;\mathrm{at}\;\mathrm{the}\;\mathrm{end}\;\mathrm{of}\;\mathrm{the}\;\mathrm{year}}$$

#### Independent variables

As health systems develop, health resources are influenced by a variety of factors, such as geographic differences (17) economic levels (37), and topographic conditions (38). Based on previous studies and data availability, we chose to explore four factors that may influence CCDC healthcare resources development in terms of social development, economic conditions, health factors, and policy factor. Table 1 shows the meaning and abbreviation of the variables.

**Table 1:** Variables and their definitions, abbreviations.

Variables	Definitions	Code
**Dependent variables**		
Disease control personnel density	the number of disease control personnel per 10,000 population	DCP_D
CCDC density	the number of CCDC per 10,000 population	CCDC_D
Health expenditures density	*per capita* health expenditures	HE_D
**Independent variables**		
Social development	Population density	PD
	Urbanization rate	UR
Economic conditions	*Per capita* total health expenditure	PTHE
	*Per capita* GDP	GDP
Health factors	Incidence of infectious diseases	IID
	Proportion of older adult population (aged over 65)	EP
Policy factor	Proportion of government finance	GF

##### Social development

Urbanization can reflect the level of public health services in a region (39). A higher level of urbanization means more developed transportation and easier access to health care services (40). The population density is used to measure the demand for healthcare resources in an area, which also benefits the government in measuring how to allocate health expenditures (41).

#### Economic conditions

*Per capita* GDP is often used as an indicator of the level of economic development (41). The distribution of human resources for health is influenced by *per capita* GDP, with higher *per capita* GDP attracting more health professionals (42). In both urban and rural areas, *per capita* health care expenditures are correlated with the amount of healthcare resources available in the neighborhood (43).

##### Health factors

Aging and infectious diseases can increase health expenditure, leading to financial burden on the country (44–46). The older adult have more and more urgent needs for healthcare services due to the deterioration of their natural physiological functions, such as reduced mobility and resistance (47). The outbreak of infectious diseases not only threatens the health of population, but also seriously affects the utilization of health care resources (42).

##### Policy factors

In China, health expenditures of the CCDC are composed of government financial input, income from healthcare services, and social funds. In fact, government finances make up a larger proportion. When the financial proportion is low, it affects the scope of health services provided by health care institutions (48).

### Methods

#### Spatial weight matrix

In this study, we use the queen collinearity matrix (49), and regions with common points or common edges are adjacent to each other. Since there is no neighboring province in Hainan Province, it is set that Hainan Province and Guangdong Province are mutually neighboring according to previous studies (50, 51). The assignment rules are as follows:


$$W_{ij}=\{\begin{array}{c} 1,\mathrm{If}\;\mathrm{there}\;\mathrm{is}\;a\;\mathrm{common}\;\mathrm{edge}\;\mathrm{or}\;\mathrm{common}\;\mathrm{point}\;\\ \mathrm{between}\;\mathrm{province}\;i\;\mathrm{and}\;\mathrm{province}\;j.\\ \;0,\mathrm{If}\;\mathrm{there}\;\mathrm{is}\;\mathrm{no}\;\mathrm{common}\;\mathrm{edge}\;\mathrm{or}\;\mathrm{common}\;\mathrm{point}\;\\ \mathrm{between}\;\mathrm{province}\;i\;\mathrm{and}\;\mathrm{province}\;j\text{.} \end{array}$$

#### Spatial autocorrelation analysis

The global Moran index and the local Moran index are usually used to analyze the spatial correlation of the studied indicators (52, 53). The global Moran index is used to characterize the spatial aggregation and distribution of overall health indicators, but it cannot clearly indicate the spatial aggregation area. Therefore, local spatial autocorrelation is used to measure the regions where spatial aggregation occurs (54). The formula is as follows:


$$\mathrm{Global}\;\mathrm{Mora}n^{\prime }s\;I=\frac{n\sum_{i=1}^{n}\sum_{j=1}^{n}W_{ij}\left(x_{i}-\bar{x}\right)\left(x_{j}-\bar{x}\right)}{\sum_{i=1}^{n}\sum_{j=1}^{n}W_{ij}\sum_{i=1}^{n}{\left(x_{i}-\bar{x}\right)}^{2}}$$

$$\mathrm{Local}\;\mathrm{Mora}n^{\prime }s\;I=\frac{n\left(x_{i}-\bar{x}\right)\sum_{j=1}^{n}W_{ij}\left(x_{j}-\bar{x}\right)}{\sum_{j=1}^{n}{\left(x_{i}-\bar{x}\right)}^{2}}$$

where $x_{i}$ and $x_{j}$ denote HP_D or CCDC_D or HE_D in provinces i and j, respectively, $\bar{x}$ is the average value, and $W_{ij}$ denotes the spatial weight matrix between provinces i and j.

The standardized statistic *z*-value is used to determine whether the global Moran index and local Moran index pass the test. The formula is as follows:


$$Z=\frac{I-E\left[I\right]}{\sqrt{\mathrm{var}\left[I\right]}}$$

where *E*[*I*] and var[*I*] are the mathematical expectation and variance, respectively.

The global Moran index has a range of values from-1 to 1. When the global Moran index I > 0, it indicates spatial positive correlation; when the global Moran index I < 0, it indicates spatial negative correlation; when I = 0, it indicates spatial random distribution. If the local Moran index >0, it means that the health indicators tend to cluster together (high values are adjacent to high values or low values are adjacent to low values), and if the local Moran index <0, it means that the health indicators do not tend to cluster together (high values are adjacent to low values or low values are adjacent to high values).

#### Spatial econometric model

Traditional OLS regression models do not consider spatial factors. Therefore, this study uses spatial econometric models to explore the factors influencing healthcare resources. There are three common traditional spatial econometric models: spatial error model (SEM), spatial lag model (SLM), and spatial Durbin model (SDM).

The SLM model is often used to explore whether the value of a spatial unit is influenced by its neighboring spaces, while the SEM model is often used to analyze the case where the dependent variable is related to a set of variables and a spatial autocorrelation error term. However, the SDM model is used to analyze the case where the dependent variable is influenced by the independent variables of this spatial unit and the neighboring spatial units. The SEM, SLM and SDM models constructed in this study are formulated as follows:

SEM:


$$\begin{array}{l} \ln Y_{\mathrm{it}}=\beta_{1}lnPD_{\mathrm{it}}+\beta_{2}lnUR_{\mathrm{it}}+\beta_{3}\mathrm{lnPTH}E_{\mathrm{it}}+\beta_{4}\mathrm{lnGD}P_{\mathrm{it}}\\ \;+\beta_{5}\mathrm{lnII}D_{\mathrm{it}}+\beta_{6}lnEP_{\mathrm{it}}+\beta_{7}lnGF_{\mathrm{it}}+\alpha +\mu_{i}+\gamma_{t}+\varepsilon_{\mathrm{it}} \end{array}$$

$$\varepsilon_{\mathrm{it}}=\lambda W\varepsilon_{\mathrm{it}}+\delta_{\mathrm{it}}$$

SLM:


$$\begin{array}{l} \ln Y_{\mathrm{it}}=\mathrm{\rho Wln}Y_{\mathrm{it}}+\beta_{1}lnPD_{\mathrm{it}}+\beta_{2}lnUR_{\mathrm{it}}+\beta_{3}\mathrm{lnPTH}E_{\mathrm{it}}+\beta_{4}\mathrm{lnGD}P_{\mathrm{it}}\\ \;+\beta_{5}\mathrm{lnII}D_{\mathrm{it}}+\beta_{6}lnEP_{\mathrm{it}}+\beta_{7}lnGF_{\mathrm{it}}+\alpha +\mu_{i}+\gamma_{t}+\varepsilon_{\mathrm{it}} \end{array}$$

SDM:


$$\begin{array}{l} \ln Y_{\mathrm{it}}=\mathrm{\rho Wln}Y_{\mathrm{it}}+\beta_{1}lnPD_{\mathrm{it}}+\beta_{2}lnUR_{\mathrm{it}}+\beta_{3}\mathrm{lnPTH}E_{\mathrm{it}}\\ \;+\beta_{4}\mathrm{lnGD}P_{\mathrm{it}}+\beta_{5}\mathrm{lnII}D_{\mathrm{it}}+\beta_{6}lnEP_{\mathrm{it}}+\beta_{7}lnGF_{\mathrm{it}}+\theta_{1}\mathrm{WlnP}D_{\mathrm{it}}\\ \;+\theta_{2}\mathrm{WlnU}R_{\mathrm{it}}+\theta_{3}\mathrm{WlnPTH}E_{\mathrm{it}}+\theta_{4}\mathrm{WlnGD}P_{\mathrm{it}}+\theta_{5}\mathrm{WlnII}D_{\mathrm{it}}\\ \;+\theta_{6}\mathrm{WlnE}P_{\mathrm{it}}+\theta_{7}\mathrm{WlnG}F_{\mathrm{it}}+\alpha +\mu_{i}+\gamma_{t}+\varepsilon_{\mathrm{it}} \end{array}$$

where $Y_{\mathrm{it}}$ denotes the dependent variable (CCDC_D, DCP_D, or HE_D) in province i in year t; *W* denotes the spatial weight matrix; ρ is the spatial autoregressive coefficient; α denotes the intercept of the regression; $\mu_{i}$ and $\gamma_{t}$ denote the individual and time effects, respectively; $\varepsilon_{\mathrm{it}}$ denotes the random error term; λ is the spatial effect of the random error; $\beta_{i}$ denotes the effect of the explanatory variable in province i on the explained variable; $\theta_{i}$ denotes the effect of the explanatory variable in neighboring provinces on the explained variable in province i.

This study uses a logarithmic transformation to reduce heteroskedasticity, implying that the effect of the independent variable on the dependent variable is explained in percentage form (55). If the correlation coefficient is less than 0.85, there is no multicollinearity in the regression model. Table 2 shows the correlations among the variables, all of which are less than 0.85, which indicates that there is no problem of multicollinearity in this study.

**Table 2:** Pearson correlation analysis between variables.

	CCDC_D	DCP_D	HE_D	lnPD	lnUR	lnPTHE	lnGDP	lnIID	lnEP	lnGF
CCDC_D	1.000									
DCP_D	0.832^***^	1.000								
HE_D	0.103	0.180^**^	1.000							
lnPD	−0.643^***^	−0.648^***^	−0.342^***^	1.000						
lnUR	−0.665^***^	−0.540^***^	0.258^***^	0.205^***^	1.000					
lnPTHE	−0.002	0.124^*^	0.584^***^	−0.299^***^	0.619^***^	1.000				
lnGDP	−0.331^***^	−0.293^***^	0.373^***^	0.105	0.807^***^	0.771^***^	1.000			
lnIID	0.444^***^	0.375^***^	−0.148^**^	−0.311^***^	−0.470^***^	−0.268^***^	−0.384^***^	1.000		
lnEP	−0.662^***^	−0.582^***^	0.030	0.534^***^	0.561^***^	0.185^**^	0.368^***^	−0.659^***^	1.000	
lnGF	0.238^***^	0.167^**^	−0.469^***^	−0.270^***^	−0.162^**^	−0.070	−0.135^*^	0.103	−0.166^**^	1.000

^*^p < 0.05; ^**^p < 0.01; ^***^p < 0.001. CCDC_D, the number of CCDC per 10,000 population; DCP_D, the number of disease control personnel per 10,000 population; HE_D, per capita health expenditures; PD, population density; UR, urbanization rate; PTHE, per capita total health expenditure; GDP, per capita GDP; IID, incidence of infectious diseases; EP, proportion of older adult population (aged over 65); GF, proportion of government finance.

To select the optimal spatial econometric model, we used the Lagrange multiplier test statistic (LM), robust Lagrange multiplier statistic (RLM), and likelihood ratio test (LR) to determine the appropriate model, and then performed the Hausman test to determine whether to choose a fixed-effects model or a random-effects model. Finally, the Goodness-of-Fit (R^2^) as the main basis for determining the model. All analyses were performed in STATA 16.0. *p* < 0.05 was considered statistically significant in this study.

## Results

### Basic information

**Figure 1:**
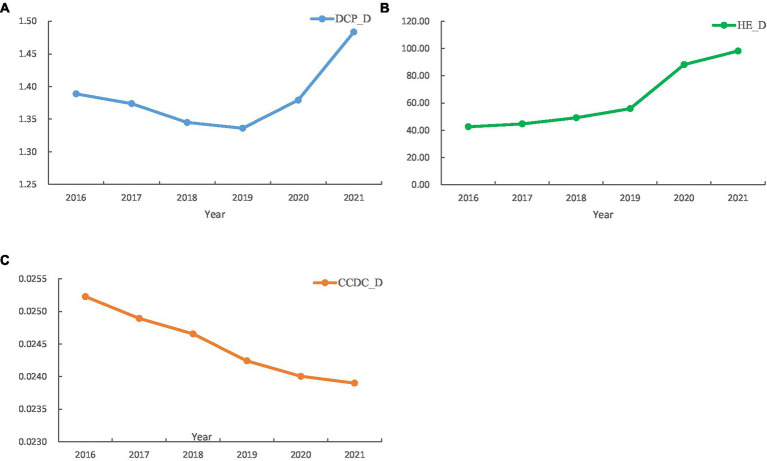
Changes of CCDC Healthcare Resources, 2016–2021.

From 2016 to 2021, DCP_D and HE_D showed an overall increasing trend, while CCDC_D showed a decreasing trend year by year (Figure 1). The descriptive statistical analysis of each dependent and independent variable in this study was shown in Table 3. The mean values of CCDC_D, DCP_D and HE_D for 2016–2021 were 1.58, 0.04 and 11.07, respectively (Table 3). It should be noted that CCDC_D and DCP_D in the western region were significantly more than those in the central and eastern regions, with Tibet having the highest CCDC_D and DCP_D. Beijing has the highest HE_D of all provinces. Compared to the policy’s disease control personnel density standards, only eight provinces qualified in 2021, and mostly located in the western region (Supplementary Table S1).

**Table 3:** Variables and descriptive statistics.

Variable	Mean	SD	Min	Max
CCDC_D	0.04	0.04	0.01	0.25
DCP_D	1.58	0.59	0.74	4.15
HE_D	11.07	18.74	0.87	113.65
PD	2653.05	1705.31	194.71	7461.18
UR	60.99	11.72	29.60	89.30
PTHE	4671.44	1934.25	2374.79	13834.01
GDP	67080.47	30743.43	27457.70	183980.00
IID	225.03	92.29	80.80	659.75
EP	11.75	2.75	5.00	17.40
GF	71.60	26.47	3.38	100.00

CCDC_D, the number of CCDC per 10,000 population; DCP_D, the number of disease control personnel per 10,000 population; HE_D, per capita health expenditures; PD, population density; UR, urbanization rate; PTHE, per capita total health expenditure; GDP, per capita GDP; IID, incidence of infectious diseases; EP, proportion of older adult population (aged over 65); GF, proportion of government finance.

### Spatial correlation analysis

**Figure 2:**
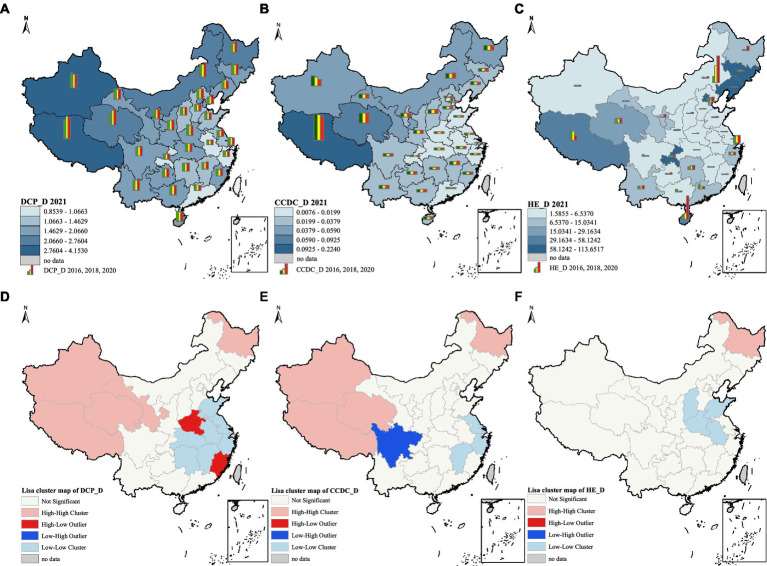
**(A)** spatial distribution of DCP_D; **(B)** spatial distribution of CCDC_D; **(C)** spatial distribution of HE_D; **(D)** univariate local indicator of spatial association cluster map of DCP_D in 2021; **(E)** univariate local indicator of spatial association cluster map of CCDC_D in 2021; **(F)** univariate local indicator of spatial association cluster map of HE_D in 2021. The average value of DCP_D, CCDC_D and HE_D in **(A–C)** was divided into five levels based on natural breaks (Jenks). These figures are drawn based on the standard map from the National Center for Basic Geographic Information (https://ngcc.cn/ngcc).

Besides HE_D and lnPD, other variables showed significant spatial autocorrelation (Tables 4, 5). The global Moran index of CCDC_D decreased from 0.1364 to 0.2662 in 6 years. However, the global Moran index of DCP_D increased from 0.4782 to 0.5067, which indicated a gradually increasing spatial autocorrelation. In addition, we mapped the spatial distribution of CCDC_D, DCP_D, and HE_D (Figures 2A–C). Overall, the three types of healthcare resources showed a trend of gradually decreasing from west to east, and the provinces with lower healthcare resources density were mainly in the eastern coastal areas.

**Table 4:** Dependent variable Moran index.

year	CCDC_D	DCP_D	HE_D
2016	0.3164^***^	0.4782^***^	0.1826^*^
2017	0.3190^***^	0.4590^***^	0.0261
2018	0.3146^***^	0.4534^***^	0.0185
2019	0.2931^***^	0.4391^***^	−0.0157
2020	0.2721^***^	0.4733^***^	0.0322
2021	0.2662^***^	0.5067^***^	0.0348

^*^p < 0.1; ^**^p < 0.05; ^***^p < 0.01. CCDC_D, the number of CCDC per 10,000 population; DCP_D, the number of disease control personnel per 10,000 population; HE_D, per capita health expenditures.

**Table 5:** Independent variable Moran index.

year	lnPD	lnUR	lnPTHE	lnGDP	lnIID	lnEP	lnGF
2016	0.1537	0.4084^***^	0.2573^***^	0.3638^***^	0.4335^***^	0.3692^***^	0.2550^**^
2017	0.1523	0.4097^***^	0.2318^**^	0.4139^***^	0.4394^***^	0.4507^***^	0.1354
2018	0.1514	0.4009^***^	0.2172^**^	0.3876^***^	0.4243^***^	0.4164^***^	0.2147^**^
2019	0.1506	0.3954^***^	0.2099^**^	0.3574^***^	0.4632^***^	0.4054^***^	0.1118
2020	0.1609^*^	0.3774^***^	0.1672^*^	0.3617^***^	0.5017^***^	0.4203^***^	−0.0239
2021	0.1633^*^	0.3767^***^	0.1671^*^	0.3574^***^	0.4628^***^	0.4203^***^	0.0996

^*^p < 0.1; ^**^p < 0.05; ^***^p < 0.01. PD, population density; UR, urbanization rate; PTHE, per capita total health expenditure; GDP, per capita GDP; IID, incidence of infectious diseases; EP, proportion of older adult population (aged over 65); GF, proportion of government finance.

The spatial agglomeration of healthcare resources density was presented as maps (Figures 2D–F). In general, high-high cluster was mainly distributed in the western and northeastern regions, including Xinjiang, Tibet, Qinghai and Heilongjiang. Eastern region (Shandong, Jiangsu and Zhejiang, etc.) and central region (Anhui, Jiangxi and Shanxi, etc.) dominated by low-low cluster.

Regarding the DCP_D, high-low cluster was mainly distributed in Henan and Fujian, while low-low cluster was mainly distributed in the central and eastern regions, and most provinces were located near the Yangtze River basin. In terms of the CCDC_D, only Sichuan showed low-high cluster, while high-high cluster was mainly distributed in the west and low-low cluster was concentrated in the east. In terms of HE_D, only a few provinces showed spatial aggregation, with Heilongjiang showing high-high cluster and the other four provinces showing low-low cluster.

### Spatial econometric analysis

The results of the statistical tests used to determine the best spatial econometric model were shown in Table 6. As an example of constructing a spatial econometric model of CCDC_D, LM (error) was significant, and the LR test judged that the SDM model would degenerate into SEM and SLM models, and the Hausman test indicated that the choice of random effects was more appropriate. In summary, the SEM model with random effects was chosen for the spatial effect analysis of the CCDC_D. Through LM test, LR test and Hausman test, we chosen the SDM model with fixed effects to analyze the spatial effects of DCP_D and HE_D.

**Table 6:** Statistical indicators of the spatial econometric model.

Test	CCDC_D	DCP_D	HE_D
Moran’s I	8.6170^***^	8.1600^***^	3.7400^***^
LM(error)	61.0690^***^	54.3970^***^	9.7820^***^
LM(lag)	3.7470^*^	24.9630^***^	0.1990
RLM(error)	66.9310^***^	29.4400^***^	14.5000^***^
RLM(lag)	9.6090^***^	0.0070	4.9170^**^
LR(lag)	10.3700	55.2000^***^	19.4400^***^
LR(error)	12.5700^*^	61.7100^***^	20.5200^***^
Hausman	9.5800	89.4800^***^	51.1400^***^

^*^p < 0.1; ^***^p < 0.01. CCDC_D, the number of CCDC per 10,000 population; DCP_D, the number of disease control personnel per 10,000 population; HE_D, per capita health expenditures.

We calculated the Goodness-of-Fit(*R*^2^) to determine individual effects, time effects, and two-way fixed effects. The bigger the *R*^2^ value, the better the model fit. As shown in Table 7, time effects were selected for both DCP_D and HE_D. Since there might be inter-individual differences among provinces over time, we chosen two-way fixed effects for further analysis of CCDC_D.

**Table 7:** The Goodness-of-Fit (*R*^2^) of the spatial econometric models.

R^2^	CCDC_D	DCP_D	HE_D
time	0.5923	0.7778	0.6712
ind	0.5923	0.2816	0.0030
both	0.5923	0.3493	0.0026

CCDC_D, the number of CCDC per 10,000 population; DCP_D, the number of disease control personnel per 10,000 population; HE_D, per capita health expenditures.

The spatial econometric models for three healthcare resources density were shown in Table 8. In the spatial error model of CCDC_D, population density and urbanization were negatively correlated with CCDC_D, with each 1% increase in population density decreasing CCDC_D by 0.03%. However, each 1% increase in infectious disease prevalence and aging increased the CCDC_D by 0.01 and 0.01%, respectively.

**Table 8:** Spatial econometric models of healthcare resources density.

variables	CCDC_D	DCP_D	HE_D
lnPD	−0.0277***	−0.1423***	−6.5272***
lnUR	−0.0392***	−2.3901***	−22.9843**
lnPTHE	0.0043	0.7234***	40.0911***
lnGDP	−0.0025	0.3103**	−4.3025
lnIID	0.0068***	0.1758*	−4.0537
lnEP	0.0082*	−0.1002	6.4237
lnGF	0.0003	0.0132	−14.6053***
WxlnPD		−0.1109	5.1469
WxlnUR		1.6467***	37.4240**
WxlnPTHE		0.1305	−8.331
WxlnGDP		−1.1561***	−11.3396
WxlnIID		−0.6465***	−5.4014
WxlnEP		−0.8721***	−20.3898
WxlnGF		0.0417	−1.5393
RE or FE	RE	FE	FE
Observations	186	186	186
R-squared	0.592	0.778	0.671
Number of provinces	31	31	31

^*^p < 0.1; ^**^p < 0.05; ^***^p < 0.01. CCDC_D, the number of CCDC per 10,000 population; DCP_D, the number of disease control personnel per 10,000 population; HE_D, per capita health expenditures; PD, population density; UR, urbanization rate; PTHE, per capita total health expenditure; GDP, per capita GDP; IID, incidence of infectious diseases; EP, proportion of older adult population (aged over 65); GF, proportion of government finance; RE, random effect; FE, fixed effect.

Since there are total, direct and indirect effects in the SDM model, the respective effects of DCP_D and HE_D are shown in Tables 9, 10. In term of DCP_D, population density, *per capita* GDP, *per capita* total health expenditure, and urbanization had different degrees of influence on local disease control personnel density. Urbanization and *per capita* total health expenditure increased by 1%, resulting in 2.38% decrease and 0.74% increase in the DCP_D in the province, respectively. In terms of spillover effects, aging, infectious disease prevalence, and *per capita* GDP increased by 1%, resulting in 1.05, 0.70, and 1.21% decrease in DCP_D in neighboring provinces, respectively. However, each 1% increase in urbanization was followed by a 1.50% increase in DCP_D in neighboring provinces (Table 9). Population density, urbanization and government financial share had negative effects on HE_D in the province, with each 1% increase decreasing HE_D in the province by 6.55, 24.18 and 14.46%, respectively. Urbanization only had significant spillover effect, with each 1% increase in local urbanization increasing the HE_D of neighboring provinces by 36.92% (Table 10).

**Table 9:** Direct, indirect, and total effects of dependent variables on DCP_D.

	Direct	Indirect	Total	Spillover effect
lnPD	−0.1448^***^	−0.1467	−0.2915^*^	No
lnUR	−2.3766^***^	1.4951^***^	−0.8816^**^	Yes
lnPTHE	0.7398^***^	0.2006	0.9404	No
lnGDP	0.2868^*^	−1.2122^***^	−0.9253^**^	Yes
lnIID	0.1416	−0.6999^***^	−0.5583^**^	Yes
lnEP	−0.1195	−1.0470^***^	−1.1665^***^	Yes
lnGF	0.0173	0.0481	0.0654	No

^*^p < 0.1; ^**^p < 0.05; ^***^p < 0.01. DCP_D, the number of disease control personnel per 10,000 population; PD, population density; UR, urbanization rate; PTHE, per capita total health expenditure; GDP, per capita GDP; IID, incidence of infectious diseases; EP, proportion of older adult population (aged over 65); GF, proportion of government finance.

**Table 10:** Direct, indirect, and total effects of dependent variables on HE_D.

	Direct	Indirect	Total	Spillover effect
lnPD	−6.5499^***^	4.8474	−1.7026	No
lnUR	−24.1832^***^	36.9194^**^	12.7362	Yes
lnPTHE	40.5638^***^	−11.4827	29.0811	No
lnGDP	−3.9678	−9.7247	−13.6925	No
lnIID	−4.7497	−5.1865	−9.9362	No
lnEP	6.9353	−22.2412	−15.3059	No
lnGF	−14.4624^***^	−1.1853	−15.6477^***^	No

^*^p < 0.1; ^**^p < 0.05; ^***^p < 0.01. HE_D, per capita health expenditures; PD, population density; UR, urbanization rate; PTHE, per capita total health expenditure; GDP, per capita GDP; IID, incidence of infectious diseases; EP, proportion of older adult population (aged over 65); GF, proportion of government finance.

## Discussion

This study aims to determine the characteristics of the spatial distribution of healthcare resources in Center for Disease Control and Prevention in China and to explore the influencing factors of health resources, which will help the health department to develop a reasonable resources allocation plan. The results showed that there was a significant spatial autocorrelation of health human resources and health physical resources, and the spatiality of health financial resources was not significant. Social, economic, policy, and health factors all had various degrees of impact on the healthcare resources of the Chinese center for disease control.

We found differences in CCDC density between provinces and the national average, in addition to marked regional differences in health expenditures density. By 2021, disease control personnel density was still below the basic requirements set by the government, with a national average disease control personnel density of 1.48 in 2021, while the government set a standard of 1.75 (56). In general, health professionals and recent medical graduates are reluctant to work at the CCDC. They prefer to work in hospitals or pharmaceutical companies with good development prospects and high salaries. This may have contributed to the shortage of health human resources in CCDC (23). In order to respond the crisis of future disease pandemics, more attention should be paid to developing the quantity and quality of disease control personnel.

This study analyzed the characteristics of the spatial distribution of disease control personnel density, CCDC density, and health expenditures density. The density of CCDC and disease control personnel showed a gradual decrease from west to east, with the highest in western regions such as Tibet, Xinjiang and Qinghai, and the lowest in eastern regions such as Shanghai, Guangdong and Jiangsu. The government of China stipulated that only one CDC should be set up in administrative districts at the county level and above (57). Western provinces such as Xinjiang and Tibet cover a wide area, and have more counties, so more CDC have been established. Moreover, the number of permanent residents in the western provinces is small, both of which make for a high CCDC density in the west. Previous studies had shown that the western region has an adequate health workforce (27, 52, 58), which was made a variety of national recruitment programs to attract talented people to work in the west (59).

However, in the developed regions of eastern China, disease control personnel density is low due to the huge local population base and the limited number of disease control personnel. The treatment of the CCDC may not match the local consumption level, leading to the flow of health talent to hospitals or companies with high salaries (60). Based on the distribution of health expenditures density, it is reasonable to infer that health expenditures are related to the severity of the epidemic in each province. In 2021, the COVID-19 epidemic is more serious in several provinces such as Tibet, Hainan and Liaoning. The government also spent significantly more on health in these provinces than in other provinces with more stable epidemics. Therefore, each province should make a strategic plan for the development of health human resources in the CCDC that is suitable for its own development, and appropriately adjust the size of CCDC personnel on the basis of the actual situation. Healthcare resources should be allocated scientifically to improve the accessibility of health services, taking into account various factors such as the number of inhabitants, geographic environment and public health events.

Spatially, the global Moran index of CCDC_D decreases, indicating that the number of CCDC gradually tends to be evenly distributed in space. Since China began a new round of health care reform in 2009, the public health system had accelerated the construction of CCDC to ensure that preventive health services are fully available (57). However, the global Moran index of disease control personnel density increased, which showed a strong spatial autocorrelation, which was similar to the results of other studies (61). Dong et al. found that the global Moran index of healthcare resources (e.g., doctors, nurses, technicians, etc.) increased to a certain extent from 2010 to 2016 (62). Cheng et al. found that the average density of health personnel increased from 1.60 in 2016 to 1.88 in 2019 (50). These studies implied a gradually expanding spatial aggregation of human resources for health. The high-high aggregation type of the density of CCDC and disease control personnel were mainly distributed in the western region, such as Xinjiang, Tibet and Qinghai, while the low-low aggregation type was distributed in the eastern region, such as Jiangxi, Zhejiang and Jiangsu. Policy changes, population mobility, and flexible employment of health workers may have contributed to this spatial aggregation. There is a necessity for the health sector to have a better understanding of the factors affecting the distribution of healthcare resources in order to be able to formulate an effective regional healthcare resources allocation policy.

In general, healthcare resources are closely linked to social development, economic conditions, policies, and so on. Urbanization, as one of the factors representing social development, has a significant impact on each of the three healthcare resources in this study. The impact of urbanization on health expenditure density is much greater than the impact on the density of CCDC and the density of disease control personnel. This may be due to that the higher the urbanization, the higher the income from health expenditures, which then has a greater impact on the density of health expenditures. In health, disease control personnel density and CCDC density are positively affected by the incidence of infectious diseases. Other scholars have also concluded that during epidemic periods, a large and well-trained health workforce is necessary to actively carry out epidemiological investigations, and so on (63).

In addition, aging has a positive effect on CCDC density. This may be due to the fact that, as the level of awareness increases and aging becomes more serious, people pay more attention to preventive health care, and local governments increase their investment in preventive health care services for the older adult population (64). At the same time, this means that the need for the CCDC is also increasing. Among the economic factors, GDP *per capita* and *per capita* health care expenditures have a positive impact on disease control personnel density, and *per capita* health care expenditures have twice the impact of GDP *per capita*. In terms of regional impacts, social development, health factors and economic factors have spillover effects on disease control personnel density in neighboring provinces. The GDP of the province is negatively correlated with the density of disease control personnel in the surrounding provinces. Each 1% increase in the province’s GDP leads to 1.21% decrease in disease control personnel density in the surrounding provinces.

Areas with higher economic levels are more likely to attract health personnel from neighboring areas who choose to work across districts (65). Particularly, as the economic level rises, it stimulates the demand for health services among the population (66), and it also increased the local demand for disease control personnel. Aging has a negative spillover effect on the density of disease control personnel in neighboring provinces. Studies have confirmed that the higher the aging, the greater the need for health workforce (67). This may be attributed to a greater need for more and more specialized health care services for the local older adult, which has led to a reduction in the number of disease control personnel in the neighboring provinces. Urbanization has a positive spillover effect on the density of disease control personnel in neighboring provinces, and we believe that urbanization has a spillover effect on health manpower, as confirmed by previous studies (68). Increasing urbanization is conducive to accelerating the mobility of human resources for health across regions. While focusing on the development of urbanization, the government should also pay attention to the impact of urbanization on healthcare resources of CCDC, and actively promote the high quality development of urbanization and CCDC.

In terms of spillover effects of health financing density, only urbanization development has a positive spillover effect on health expenditures density. Increasing urbanization in the province by 1% will cause the health expenditures density in the surrounding provinces to increase by 36.92%. It can be understood that the better developed the city, the higher the level of the health economy, causing the government to redirect investment to neighboring poorer provinces.

This study also has some limitations. Firstly, we calculated healthcare resources density based on population size only and conducted a study on spatial distribution and influencing factors. In the next study, healthcare resources density can be calculated based on land area. Secondly, other factors such as education levels and salary levels may have an impact on healthcare resources (69, 70). However, due to the availability of data, it was not possible to include the level of education and salary level in the study of influencing factors.

## Conclusion

In conclusion, our study identified significant spatial variation in healthcare resources allocation of CDC in China. Moreover, the spatial aggregation of health human and health financial resources increased positively over the study period, which reflects the expansion of spatial variability. Social development, economic conditions, health factors and policy factors had impacts on the allocation of healthcare resources not only in the province but also in neighboring provinces.

## Data availability statement

Publicly available datasets were analyzed in this study. This data can be found at: https://www.stats.gov.cn/.

## Author contributions

YiY: Writing – original draft, Writing – review & editing. JL: Writing – review & editing. XD: Writing – review & editing. YaY: Writing – review & editing. LZ: Writing – review & editing.

## References

[1] Qiu L Yang L Li H Wang L. The impact of health resource enhancement and its spatiotemporal relationship with population health. Front Public Health. (2023) 10:10. doi: 10.3389/fpubh.2022.1043184 PMC9868711 36699901

[2] Wang Z He H Liu X Wei H Feng Q Wei B. Health resource allocation in Western China from 2014 to 2018. Arch Public Health. (2023) 81:30. doi: 10.1186/s13690-023-01046-x, PMID: 36814309 PMC9946701

[3] Hou J Ke Y. Addressing the shortage of health professionals in rural China: issues and progress comment on have health human resources become more equal between rural and urban areas after the new reform? Int J Health Policy Manag. (2015) 4:327–8. doi: 10.15171/ijhpm.2015.57, PMID: 25905487 PMC4417640

[4] Chen Y Yin Z Xie Q. Suggestions to ameliorate the inequity in urban/rural allocation of healthcare resources in China. Int J Equity Health. (2014) 13:34. doi: 10.1186/1475-9276-13-34, PMID: 24884614 PMC4016733

[5] Farahani M Subramanian SV Canning D. The effect of changes in health sector resources on infant mortality in the short-run and the long-run: a longitudinal econometric analysis. Soc Sci Med. (2009) 68:1918–25. doi: 10.1016/j.socscimed.2009.03.023, PMID: 19362762

[6] Meyer SP. Comparing spatial accessibility to conventional medicine and complementary and alternative medicine in Ontario. Canada Health Place. (2012) 18:305–14. doi: 10.1016/j.healthplace.2011.10.005, PMID: 22088264

[7] Marwali EM Kekalih A Yuliarto S Wati DK Rayhan M Valerie IC. Paediatric COVID-19 mortality: a database analysis of the impact of health resource disparity. BMJ Paediatr Open. (2022) 6:e001657. doi: 10.1136/bmjpo-2022-001657, PMID: 36645791 PMC9621167

[8] Lisboa M Fronteira I Mason PH Martins MRO. National TB program shortages as potential factor for poor-quality TB care cascade: healthcare workers’ perspective from Beira, Mozambique. PLoS One. (2020) 15:e0228927. doi: 10.1371/journal.pone.0228927, PMID: 32059032 PMC7021283

[9] Bartsch SM Ferguson MC McKinnell JA O'Shea KJ Wedlock PT Siegmund SS. The potential health care costs and resource use associated with COVID-19 in the United States. Health Aff. (2020) 39:927–35. doi: 10.1377/hlthaff.2020.00426, PMID: 32324428 PMC11027994

[10] Pinho M Araújo A. *How to fairly allocate scarce medical resources?* Controversial preferences of healthcare professionals with different personal characteristics. Health Economics, Policy and Law, pp. 1–18. (2021). 10.1017/S1744133121000190 34108069

[11] Kodama S Uwatoko F Koriyama C. Relationship between changes in the public health nurses’ workforce and the empirical Bayes estimates of standardized mortality ratio: a longitudinal ecological study of municipalities in Japan. BMC Health Serv Res. (2023) 23:266. doi: 10.1186/s12913-023-09273-2, PMID: 36932374 PMC10022064

[12] Noor AM Ngugi AK Agoi F Mahoney MR Lakhani A Mang’ong’o D. Utilization of health services in a resource-limited rural area in Kenya: prevalence and associated household-level factors. PLoS One. (2017) 12:728. doi: 10.1371/journal.pone.0172728 PMC5328402 28241032

[13] Kanchanachitra C Lindelow M Johnston T Hanvoravongchai P Lorenzo FM Huong NL. Human resources for health in Southeast Asia: shortages, distributional challenges, and international trade in health services. Lancet. (2011) 377:769–81. doi: 10.1016/S0140-6736(10)62035-1, PMID: 21269674

[14] Kaur G Prinja S Lakshmi PVM Downey L Sharma D Teerawattananon Y. Criteria used for priority-setting for public health resource allocation in low-and middle-income countries: a systematic review. Int J Technol Assess Health Care. (2019) 35:474–83. doi: 10.1017/S0266462319000473, PMID: 31307561

[15] Liu W Liu Y Twum P Li S. National equity of health resource allocation in China: data from 2009 to 2013. Int J Equity Health. (2016) 15:68. doi: 10.1186/s12939-016-0357-1, PMID: 27093960 PMC4837535

[16] Wang X Luo H Qin X Feng J Gao H Feng Q. Evaluation of performance and impacts of maternal and child health hospital services using data envelopment analysis in Guangxi Zhuang autonomous region, China: a comparison study among poverty and non-poverty county level hospitals. Int J Equity Health. (2016) 15:131. doi: 10.1186/s12939-016-0420-y, PMID: 27552805 PMC4994280

[17] Wang X Yang H Duan Z Pan J. Spatial accessibility of primary health care in China: a case study in Sichuan Province. Soc Sci Med. (2018) 209:14–24. doi: 10.1016/j.socscimed.2018.05.023, PMID: 29778934

[18] Tong MX Hansen A Hanson-Easey S Xiang J Cameron S Liu Q. Public health professionals’ perceptions of the capacity of China’s CDCs to address emerging and re-emerging infectious diseases. J Public Health. (2021) 43:209–16. doi: 10.1093/pubmed/fdz070, PMID: 31251367

[19] Li C Sun M Shen JJ Cochran CR Li X Hao M. Evaluation on the efficiencies of county-level Centers for Disease Control and Prevention in China: results from a national survey. Trop Med Int Health. (2016) 21:1106–14. doi: 10.1111/tmi.12753, PMID: 27404084

[20] China SCotCPo. *Outline of the "healthy China 2030" plan 2016*. (2016). Available at: https://www.gov.cn/zhengce/2016-10/25/content_5124174.htm.

[21] Luo Li WY Tiantian Z. Thoughts on the construction of disease prevention and control system in the new era. Chin Health Resources. (2020) 23:7–13. doi: 10.13688/j.cnki.chr.2020.19226

[22] Wei X. *Measures to shore up disease control and prevention capacities: CHINADAILY*. (2024). Available at: https://www.chinadaily.com.cn/a/202401/03/WS65949e1ca3105f21a507a372.html.

[23] Li YQ Chen H Guo HY. Examining inequality in the public health workforce distribution in the Centers for Disease Control and Prevention (CDCs) system in China, 2008-2017. Biomed Environ Sci. (2020) 33:374–83. doi: 10.3967/bes2020.051, PMID: 32553083

[24] Chen Cheng ZN Ting C. Spatial analysis of the staffing level of Centers for Disease Control and Prevention in China. Chin J Health Policy. (2021) 14:58–65. doi: 10.3969/j.issn.1674-2982.2021.06.009

[25] Li Liping ZX Zhao Yulan L Zuxun Z. Degree of coordination between primary care resource allocation and economic development in eastern, central and Western China. Chin. Gen Pract. (2021) 24:2777–84. doi: 10.12114/j.issn.1007-9572.2021.00.234

[26] Love-Koh J Griffin S Kataika E Revill P Sibandze S Walker S. Methods to promote equity in health resource allocation in low-and middle-income countries: an overview. Glob Health. (2020) 16:6. doi: 10.1186/s12992-019-0537-z, PMID: 31931823 PMC6958737

[27] Ao Y Feng Q Zhou Z Chen Y Wang T. Resource allocation equity in the China’s rural three-tier healthcare system. Int J Environ Res Public Health. (2022) 19:589. doi: 10.3390/ijerph19116589, PMID: 35682174 PMC9180023

[28] Sun J. Equality in the distribution of health material and human resources in Guangxi: evidence from southern China. BMC Res Notes. (2017) 10:2760. doi: 10.1186/s13104-017-2760-0 PMC5576300 28851453

[29] Lu L Zeng J. Inequalities in the geographic distribution of hospital beds and doctors in traditional Chinese medicine from 2004 to 2014. Int J Equity Health. (2018) 17:165. doi: 10.1186/s12939-018-0882-1, PMID: 30419919 PMC6233493

[30] Yan K Chun-Li C Jia-Ni WU Yi-Feng Z Shi-Zhu LI Xiao-Nong Z. General status and research progress of human resource in the institutions of disease control and prevention in China. Chin. J. Public Health Manag. (2017) 33:334–8. doi: 10.19568/j.cnki.23-1318.2017.03.014

[31] Yao Min MH Lidong G Fuqiang L Qun Z Juan Z. Analysis of the status of health human resources allocation in Hunan Province's disease control institutions. Pract Prev Med. (2020) 27:11. doi: 10.3969/j.issn.1006-3110.2020.09.033

[32] Qiongwenqian W Lieyu H Yang L Yan G. Current situation and suggestions on the construction of talent team in the institutions of disease control and prevention in China. Chinese journal of public health. Management. (2021) 37:165–8. doi: 10.19568/j.cnki.23-1318.2021.02.0007

[33] Shan Ying LG Shixue L. A temporal and spatial analysis of the staffing allocation level of China's centers for disease and prevention from 2009 to 2019. Chinese. Health Econ. (2022) 41:46–50. doi: 10.7664/j.issn.1003-0743.2022.3.zgwsjj202203011

[34] Chai K-C Zhang Y-B Chang K-C. Regional disparity of medical resources and its effect on mortality rates in China. Front Public Health. (2020) 8:8. doi: 10.3389/fpubh.2020.00008, PMID: 32117848 PMC7011092

[35] Panopoulou E Pantelidis T. Convergence in per capita health expenditures and health outcomes in the OECD countries. Appl Econ. (2012) 44:3909–20. doi: 10.1080/00036846.2011.583222

[36] Railaite R Čiutienė R. The impact of public health expenditure on health component of human capital. Eng Econ. (2020) 31:371–9. doi: 10.5755/j01.ee.31.3.25158

[37] Bhattacharjee A Maiti T Petrie D. General equilibrium effects of spatial structure: health outcomes and health behaviours in Scotland. Reg Sci Urban Econ. (2014) 49:286–97. doi: 10.1016/j.regsciurbeco.2014.10.003

[38] Wu X Mao R Guo X. Equilibrium of tiered healthcare resources during the COVID-19 pandemic in China: a case study of Taiyuan, Shanxi Province. Int J Environ Res Public Health. (2022) 19:35. doi: 10.3390/ijerph19127035 PMC9222232 35742282

[39] Haixiang L. Research on the health risk of public governance in the process of urbanization - based on spatial vision. J Shanghai Univ Int Bus Econ. (2020) 27:102–11. doi: 10.16060/j.cnki.issn2095-8072.2020.03.009

[40] Zou Y Jia L Chen S Deng X Chen Z He Y. Spatial accessibility of emergency medical services in Chongqing, Southwest China. Front Public Health. (2023) 10:10. doi: 10.3389/fpubh.2022.959314 PMC9853430 36684945

[41] Liu T Li J Chen J Yang S. Regional differences and influencing factors of allocation efficiency of rural public health resources in China. Healthcare. (2020) 8:270. doi: 10.3390/healthcare8030270, PMID: 32823864 PMC7551190

[42] Carvalho Rde S Diniz AS Lacerda FM Mello PA. Gross domestic product (GDP) per capita and geographical distribution of ophthalmologists in Brazil. Arq Bras Oftalmol. (2012) 75:407–11. doi: 10.1590/S0004-27492012000600007, PMID: 23715143

[43] Song X Wei Y Deng W Zhang S Zhou P Liu Y. Spatio-temporal distribution, spillover effects and influences of China’s two levels of public healthcare resources. Int J Environ Res Public Health. (2019) 16:582. doi: 10.3390/ijerph16040582, PMID: 30781583 PMC6407009

[44] Tang B Li Z. A country-level empirical study on the fiscal effect of elderly population health: the mediating role of healthcare resources. Healthcare. (2021) 10:30. doi: 10.3390/healthcare10010030, PMID: 35052194 PMC8775273

[45] Pammolli F Riccaboni M Magazzini L. The sustainability of European health care systems: beyond income and aging. Eur J Health Econ. (2011) 13:623–34. doi: 10.1007/s10198-011-0337-8, PMID: 21814838

[46] Bloom DE Cadarette D. Infectious disease threats in the twenty-first century: strengthening the global response. Front Immunol. (2019) 10:549. doi: 10.3389/fimmu.2019.00549 30984169 PMC6447676

[47] Guida C Carpentieri G. Quality of life in the urban environment and primary health services for the elderly during the Covid-19 pandemic: an application to the city of Milan (Italy). Cities. (2021) 110:103038. doi: 10.1016/j.cities.2020.103038, PMID: 33262550 PMC7691131

[48] Li Z Hung P He R Zhang L. Association between direct government subsidies and service scope of primary care facilities: a cross-sectional study in China. Int J Equity Health. (2020) 19:135. doi: 10.1186/s12939-020-01248-7, PMID: 32778111 PMC7418383

[49] Smiley MJ Diez Roux AV Brines SJ Brown DG Evenson KR Rodriguez DA. A spatial analysis of health-related resources in three diverse metropolitan areas. Health Place. (2010) 16:885–92. doi: 10.1016/j.healthplace.2010.04.014, PMID: 20478737 PMC3297427

[50] Chen C Chen T Zhao N Dong S. Regional maldistribution of human resources of rehabilitation institutions in China mainland based on spatial analysis. Front Public Health. (2022) 10:1028235. doi: 10.3389/fpubh.2022.1028235 36424956 PMC9679792

[51] Chen B Jin F. Spatial distribution, regional differences, and dynamic evolution of the medical and health services supply in China. Front Public Health. (2022) 10:10. doi: 10.3389/fpubh.2022.1020402 PMC9540227 36211684

[52] Zhu B Fu Y Liu J He R Zhang N Mao Y. Detecting the priority areas for health workforce allocation with LISA functions: an empirical analysis for China. BMC Health Serv Res. (2018) 18:957. doi: 10.1186/s12913-018-3737-y, PMID: 30541543 PMC6292090

[53] Chen J Lin Z Li L-a Li J Wang Y Pan Y. Ten years of China’s new healthcare reform: a longitudinal study on changes in health resources. BMC Public Health. (2021) 21:2272. doi: 10.1186/s12889-021-12248-9, PMID: 34903184 PMC8670033

[54] Anselin L. Local indicators of spatial association-LISA. Geogr Anal. (2010) 27:93–115. doi: 10.1111/j.1538-4632.1995.tb00338.x

[55] Zhu B Hsieh C-W Mao Y. Addressing the licensed doctor maldistribution in China: a demand-and-supply perspective. Int J Environ Res Public Health. (2019) 16:1753. doi: 10.3390/ijerph16101753, PMID: 31108920 PMC6571941

[56] Institute SIoCMR. *Suggestions on accelerating the allocation of public health resources* (2020). Available at: https://www.ndrc.gov.cn/xxgk/jd/wsdwhfz/202012/t20201218_1253062.html.

[57] China GOotSCo. *National health service system planning outline*. (2015). Available at: https://www.gov.cn/zhengce/content/2015-03/30/content_9560.htm.

[58] Zhu B Hsieh C-W Zhang Y. Incorporating spatial statistics into examining equity in health workforce distribution: an empirical analysis in the Chinese context. Int J Environ Res Public Health. (2018) 15:1309. doi: 10.3390/ijerph15071309, PMID: 29932139 PMC6068954

[59] CPC Central Committee SC. *Guiding opinions on promoting the development of the western region to form a new pattern in the new era 2020*. Available at: http://www.xinhuanet.com/politics/zywj/2020-05/17/c_1125996720.htm.

[60] Liu S Gu Y Yang Y Schroeder E Chen Y. Tackling brain drain at Chinese CDCs: understanding job preferences of public health doctoral students using a discrete choice experiment survey. Hum Resour Health. (2022) 20:46. doi: 10.1186/s12960-022-00743-y, PMID: 35606873 PMC9125964

[61] Bai Q Ke X Huang L Liu L Xue D Bian Y. Finding flaws in the spatial distribution of health workforce and its influential factors: an empirical analysis based on Chinese provincial panel data, 2010–2019. Front Public Health. (2022) 10:10. doi: 10.3389/fpubh.2022.953695 PMC9794860 36589992

[62] Dong E Sun X Xu T Zhang S Wang T Zhang L. Measuring the inequalities in healthcare resource in facility and workforce: a longitudinal study in China. Front Public Health. (2023) 11:11. doi: 10.3389/fpubh.2023.1074417 PMC10060654 37006575

[63] Kilmarx PH Long T Reid MJA. A National Public Health Workforce to control COVID-19 and address health disparities in the United States. Open Forum Infect Dis. (2021) 8:304. doi: 10.1093/ofid/ofab304 PMC8271123 34258323

[64] Cristea M Noja GG Stefea P Sala AL. The impact of population aging and public health support on EU labor markets. Int J Environ Res Public Health. (2020) 17:439. doi: 10.3390/ijerph17041439, PMID: 32102277 PMC7068414

[65] Yang Y Zhang L Zhang X Yang M Zou W. Efficiency measurement and spatial spillover effect of provincial health systems in China: based on the two-stage network DEA model. Front Public Health. (2022) 10:952975. doi: 10.3389/fpubh.2022.952975 36262222 PMC9574077

[66] Jakovljevic M Timofeyev Y Ranabhat CL Fernandes PO Teixeira JP Rancic N. Real GDP growth rates and healthcare spending – comparison between the G7 and the EM7 countries. Glob Health. (2020) 16:64. doi: 10.1186/s12992-020-00590-3, PMID: 32677998 PMC7367257

[67] Sellers K Leider JP Gould E Castrucci BC Beck A Bogaert K. The state of the US governmental public health workforce, 2014–2017. Am J Public Health. (2019) 109:674–80. doi: 10.2105/AJPH.2019.305011, PMID: 30896986 PMC6459653

[68] Wang L Chen Y. Determinants of China’s health expenditure growth: based on Baumol’s cost disease theory. Int J Equity Health. (2021) 20:213. doi: 10.1186/s12939-021-01550-y, PMID: 34565389 PMC8474747

[69] Ravangard R Hatam N Teimourizad A Jafari A. Factors affecting the technical efficiency of health systems: a case study of economic cooperation organization (ECO) countries (2004–10). Int J Health Policy Manag. (2014) 3:63–9. doi: 10.15171/ijhpm.2014.60, PMID: 25114944 PMC4122076

[70] Asante AD Zwi AB. Factors influencing resource allocation decisions and equity in the health system of Ghana. Public Health. (2009) 123:371–7. doi: 10.1016/j.puhe.2009.02.006, PMID: 19364613

