# Supplementary material for: Spatial distribution and influencing factors of CDC health resources in China: a study based on panel data from 2016–2021
Source: Front Public Health. 2024 May 1;12:1331522. doi: 10.3389/fpubh.2024.1331522 (PMC11094321; doi:10.3389/fpubh.2024.1331522)
Supplement: Supplementary file 1 [file Table_1.DOCX]

Supplementary Material

Spatial distribution and influencing factors of CDC health resources in China: a study based on panel data from 2016-2021

Yingying Yu, Jiachen Lu, Xiaofeng Dou, Yaohui Yi and Ling Zhou*

*** Correspondence: Ling Zhou:** [**zhouling0609@163.com**](mailto:zhouling0609@163.com)

Supplementary Table S1 Health resource density in 31 provinces in China in 2021.

| Region | Province | CDC_D | HP_D | HE_D |
| --- | --- | --- | --- | --- |
| Overall |  | 0.02 | 1.48 | 14.66 |
| Eastern region | Beijing | 0.01 | 1.63 | 113.65 |
|  | Tianjin | 0.01 | 1.69 | 29.16 |
|  | Hebei | 0.03 | 1.24 | 1.59 |
|  | Liaoning | 0.03 | 1.56 | 82.65 |
|  | Shanghai | 0.01 | 1.32 | 58.12 |
|  | Jiangsu | 0.01 | 1.29 | 2.07 |
|  | Zhejiang | 0.02 | 0.97 | 11.53 |
|  | Fujian | 0.02 | 1.65 | 5.54 |
|  | Shandong | 0.02 | 1.30 | 4.06 |
|  | Guangdong | 0.01 | 0.85 | 9.49 |
|  | Hainan | 0.03 | 1.60 | 43.89 |
| Western region | Inner Mongolia | 0.05 | 2.76 | 6.04 |
|  | Guangxi | 0.02 | 1.61 | 6.54 |
|  | Chongqing | 0.01 | 1.07 | 96.88 |
|  | Sichuan | 0.03 | 1.67 | 3.08 |
|  | Guizhou | 0.03 | 1.60 | 5.02 |
|  | Yunnan | 0.03 | 2.07 | 10.96 |
|  | Tibet | 0.22 | 4.15 | 40.77 |
|  | Shaanxi | 0.03 | 1.67 | 3.52 |
|  | Gansu | 0.04 | 1.92 | 7.90 |
|  | Qinghai | 0.09 | 2.58 | 18.56 |
|  | Ningxia | 0.03 | 1.65 | 7.98 |
|  | Xinjiang | 0.06 | 3.76 | 2.84 |
| Central region | Shanxi | 0.04 | 1.46 | 2.16 |
|  | Jilin | 0.03 | 1.92 | 84.73 |
|  | Heilongjiang | 0.05 | 2.18 | 12.14 |
|  | Anhui | 0.02 | 0.94 | 2.01 |
|  | Jiangxi | 0.03 | 1.27 | 4.47 |
|  | Henan | 0.02 | 1.69 | 2.57 |
|  | Hubei | 0.02 | 1.54 | 3.22 |
|  | Hunan | 0.02 | 1.46 | 15.03 |

CDC_D: health personnel density per 10,000 population; HP_D: density of CDC per 10,000 population; HE_D: health expenditure density per capita.
